# Shielding device for endoscopic procedures during lower gastrointestinal endoscopy

**DOI:** 10.1002/deo2.173

**Published:** 2022-10-10

**Authors:** Daisuke Kikuchi, Daiki Ariyoshi, Yugo Suzuki, Yorinari Ochiai, Hiroyuki Odagiri, Junnosuke Hayasaka, Masami Tanaka, Tetsuya Morishima, Keita Kimura, Hiroshi Ezawa, Sanae Nakagawa, Risa Iwamoto, Yoshinori Matsuwaki, Shu Hoteya

**Affiliations:** ^1^ Department of Gastroenterology Toranomon Hospital Tokyo Japan; ^2^ Olympus Medical Systems Corporation Tokyo Japan; ^3^ Olympus Corporation Tokyo Japan; ^4^ Matsuwaki Clinic Shinagawa Tokyo Japan

**Keywords:** aerosol infection, colonoscopy, contact infection, coronavirus, droplet infection

## Abstract

**Objectives:**

The coronavirus pandemic significantly impacted endoscopic practice. During lower gastrointestinal endoscopy, infectious substances disseminate; therefore, we developed an infection control device (STEP‐L) for lower gastrointestinal endoscopy and examined its usefulness.

**Methods:**

STEP‐L wraps around the patient's buttocks and covers the endoscope. Using lower endoscopy training models, three endoscopists performed 18 colonoscopies with STEP‐L (group S) and without (group C). Endoscopic insertion time and pigmented areas of ​​gloves and diapers after the examination were compared between both groups.

**Results:**

Insertion of the endoscope up to the cecum was possible in all 18 examinations. The insertion time to the cecum was 52.4 ± 19.0 s in group S and 53.9 ± 13.3 s in group C. The pigmented areas of the ​​gloves measured 39,108.0 ± 16,155.3 pixels in group C, but were significantly reduced to 2610.5 ± 4333.8 pixels in group S (*p* < 0.05). The pigmented areas of the diapers measured 2280.9 ± 3285.2 pixels in group C, but were significantly reduced to 138.0 ± 82.9 pixels in group S (*p* < 0.05).

**Conclusions:**

Using STEP‐L does not change the insertion time, and is technically feasible. STEP‐L significantly reduces the adhesion of virtual pollutants to the surroundings, suggesting that this device is useful for infection control during lower gastrointestinal endoscopy.

## INTRODUCTION

Since the novel coronavirus disease 2019 (COVID‐19) was reported in 2019, cases have been reported worldwide. The lockdown of cities due to the COVID‐19 pandemic has had a major impact on the economy. The pandemic has also significantly affected endoscopic practice, and the number of endoscopic examinations has temporarily decreased significantly. Guidelines to prevent the spread of the infection during gastrointestinal endoscopy have been reported in many academic societies.^1^, ^2^, ^3^ Endoscopists currently have to decide whether to perform endoscopy considering the possibility of COVID‐19 infection and the need for endoscopy. In addition, current personal protective equipment (PPE) is divided according to the level of risk of infection.^4^

During lower gastrointestinal endoscopy (LGE), there is a risk of the spread of various infections due to the diffusion of intestinal juice and stool into the surrounding environment. We are currently developing a new shielding device (shielding device for lower gastrointestinal endoscopic procedures: STEP‐L) in collaboration with Olympus for cleaner clinical practice during LGE. In this study, we aimed to assess the feasibility of using this device and assess its effectiveness in preventing the spread of infectious materials.

## MATERIAL AND METHODS

### Device

**FIGURE 1:**
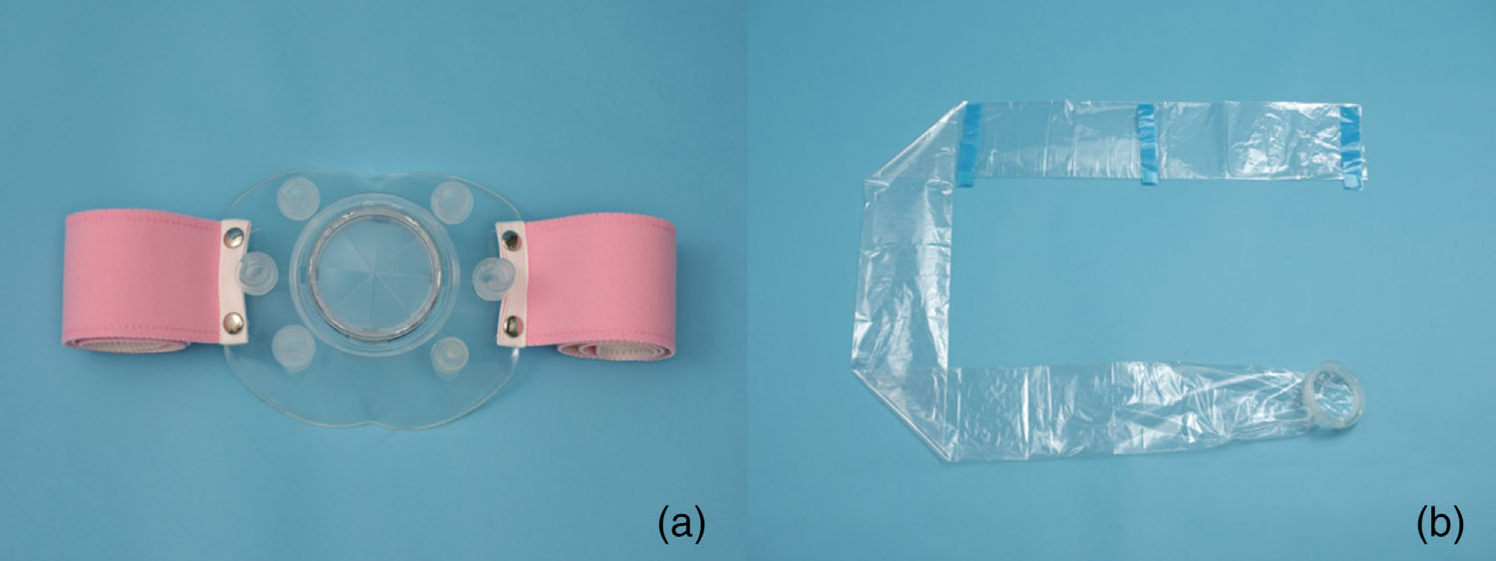
Shielding device for lower gastrointestinal endoscopy. STEP‐L consists of a pad (Figure 1a) that is attached to the patient's buttocks and a drape (Figure 1b) that is attached to the endoscope

A device for the prevention of contact infection during LGE (STEP‐L) was developed in collaboration with Olympus Medical Systems. STEP‐L consists of a pad that is attached to the patient's buttocks and a drape that is attached to the endoscope (Figure 1). The pad contains a hole for inserting an endoscope and a band that is wrapped around the patient's buttocks in order to fix it. The total length of the drape is 200 cm. The pad and drape are connected by a connecting section with a one‐touch lock mechanism.

### How to use

**FIGURE 2:**
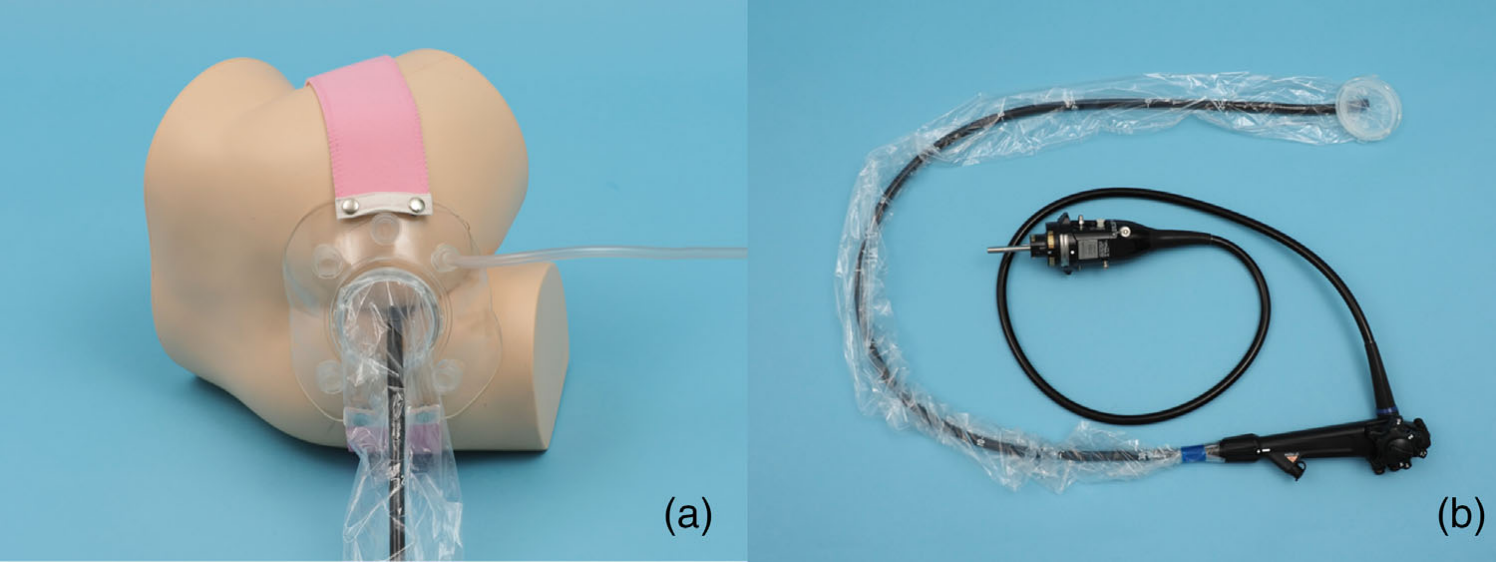
Image when STEP‐L is attached to the endoscope and the patient's buttocks. The pad is placed on the patient's buttocks and fixed to the buttocks with a band. (Figure 2a). The drape portion is fixed to the base of the endoscope with tapes, and the endoscope is covered with the drape (Figure 2b)

First, the drape portion is fixed to the base of the endoscope with tapes, and the endoscope is covered with the drape (Figure 2). Since the drape is 200 cm, it is cut or folded according to the length of the endoscope to be used. The pad is then placed on the patient's buttocks and fixed to the buttocks with a band. The patient is placed on the examination bed in the left‐lateral position. The pad and the drape parts are connected to the connection part. The endoscopist operates the endoscope through the drape, and after the endoscopic examination, the drape is then detached from the pad. The endoscope is then handed to the assistant while it is covered with a drape, and the examination is completed.

## EXPERIMENT

We conducted an experiment to verify the feasibility of using STEP‐L. In this study, we used a colonoscopy insertion training model (CM‐15; Kyoto Kagaku Co., Ltd., Kyoto, Japan). The endoscope used was EVIS LUCERA ELITE COLONOVIDEOSCOPE PCF‐H290I (Olympus Medical Systems Corp., Tokyo, Japan). Three endoscopists performed colonoscopy 18 times; three times each with and without STEP‐L. Two of the participating endoscopists were qualified specialists of the Japanese Gastroenterological Endoscopy Society. All endoscopists in this study have experienced more than 1000 cases of colonoscopy. The insertion time from the anus to the cecum between the group with STEP‐L (S group) and the group without (conventional group: C group) was compared.

**FIGURE 3:**
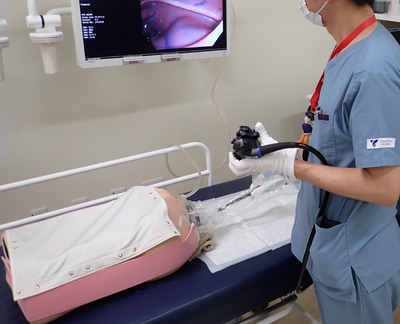
Actual image of the experiment in this study. The pigmented area of the white glove and diaper were evaluated

Indigo carmine was applied to the entire colon. Before each endoscopist's examination, colonoscopy was inserted into the cecum and indigo carmine was sprayed on the entire colon using a spray tube. A white diaper was laid under the model, and the endoscopist wore a white glove. In total, 18 insertions and observations were performed with and without STEP‐L. At the time of observation, the cecum, hepatic flexure, right side, middle, and left side of the transverse colon, splenic flexure, descending colon, sigmoid colon, and rectum were photographed (Figure 3). After the examination, the endoscopists’ gloves on the right hand and the diapers were scanned, the pigmentation site was measured using Image J (US National Institutes of Health, Bethesda, Maryland, USA), and the number of pixels of the pigmentation sites in both groups was compared.

### Statistical analysis

Data were analyzed using the unpaired *t*‐test, chi‐squared test, Fisher's test, or Mann‐Whitney U‐test, as appropriate. A *p*‐value of <0.05 indicated statistical significance. All statistical analyses were performed using IBM SPSS Statistics for Windows, version 20.0 (IBM Corp., Armonk, NY, USA).

## RESULTS

In all examinations, insertion into the cecum was possible. The insertion time was 52.4 ± 19.0 s in group S and 53.9 ± 13.3 s in group C.

The area of pigmentation on the gloves measured 39108.0 ± 16155.3 pixels in the C group, but was significantly reduced to 2610.5 ± 4333.8 pixels in the S group (*p* < 0.05). The area of pigmentation on the diapers measured 2280.9 ± 3285.2 pixels in group C, but was significantly reduced to 138.0 ± 82.9 pixels in group S (*p* < 0.05).

## DISCUSSION

As of May 2021, more than 170 million cases of COVID‐19 infection had been reported worldwide. Endoscopic practice is associated with the risk of contact with infectious droplets and aerosols, and many academic societies have proposed various recommendations. In addition, various methods for reducing infections during endoscopic procedures have been reported.^5^, ^6^, ^7^, ^8^, ^9^, ^10^ Most reports are on methods of shielding endoscopists from the patient's cough and vomitus. Therefore, most reports are on upper gastrointestinal endoscopy, but there are few reports on measures for lower endoscopy.

The risk of infection during upper gastrointestinal endoscopy is not limited to contact infection, but droplets and aerosols are also major problems. Sagami et al. measured the aerosol in upper gastrointestinal endoscopy and reported that the amount of aerosol produced was higher in people with a higher body mass index (BMI) and in those who belched during the examination compared to those with a lower BMI and those who did not belch.^11^ However, the main route of infection spread during LGE is contact. It is considered that LGE produces fewer droplets and aerosols from the patient compared to those produced during upper gastrointestinal endoscopy. However, even in LGE, there is a risk of contact transmission of various infectious diseases by touching the contaminated endoscope. Recently, it has also been reported that droplets and aerosols are generated during LGE.^12^, ^13^ For this reason, further infection control is necessary, and we have developed a new device this time.

The use of this device reduces the possibility of direct contact with a contaminated endoscope. It can also reduce the possibility of the contaminated endoscope coming into contact with the surrounding environment. In this experiment, pigmentations of the endoscopist's gloves and diapers on the examination bed were significantly lower in the S group than in the C group, suggesting that it may be useful for infection control in LGE. In addition, there is a possibility that the risk of contact with contaminants can be reduced when transporting the endoscope after use. It may be useful not only for COVID‐19 countermeasures but also for creating a cleaner environment during the endoscopic perioperative period. However, in this study, a small number of contaminants were observed even in the S group, and it is considered essential to use conventional PPE.

This study has some limitations. The first is the structure of the pad. The newly developed product can be fixed for patients with different body shapes by forming a band‐shaped band. However, depending on the patient, the adhesion between the pad and the patient's buttocks is poor, and intestinal juice may flow out to the examination bed. In the future, we would like to improve the shape and material of the pad to improve adhesion. A clinical randomized controlled trial is currently underway to verify the usefulness of this device. In this clinical trial, the influence of this device on endoscopic maneuverability and insertion is verified. In addition, this time, we did not evaluate the infection from the forceps channel in this experiment. However, it has been reported that there is backflow from the forceps channel when using a laryngoscope,^14^ and it is necessary to investigate colonoscopy in the future. Another limitation is that the experiment had a small number of training models. More endoscopists will need to positively verify the effectiveness of this device. In particular, evaluation according to the skill of the endoscopist is considered necessary. Moreover, in this experiment, the insertion times were compared and the results were similar. However, the effect on observation time is also important. In our ongoing clinical trial, observation time and pain levels of the patients are verified and the results will be reported in the future.

In conclusion, we developed a dedicated device to prevent contact infection during LGE. Contaminated endoscopes do not come into contact with the surroundings because they operate through a shield. During verification using the training model, the operability of the endoscope was not particularly affected, and the diffusion of surrounding pollutants was reduced. It is necessary to verify the feasibility and utility of this device in clinical studies, and we suggest that this device may be useful in reducing the spread of infections during LGE (Table 1).

**TABLE 1:** Results of the study

	**Group C**	**Group S**	***p*‐value**
Insertion rate (%, *n*/*n*)	100 (9/9)	100 (9/9)	N.S.
Insertion time (sec ± SD)	53.9 ± 13.3	52.4 ± 19.0	N.S.
Pigmented area of the gloves (pixels ± SD)	39108.0 ± 16155.3	2610.5 ± 4333.8	<0.05
Pigmented area of the diapers (pixels ± SD)	2280.9 ± 3285.2	138.0 ± 82.9	<0.05

## CONFLICT OF INTEREST

This research and development were carried out in collaboration with Olympus Medical Systems Corporation. Toranomon Hospital and Olympus Medical Systems Corporation have entered into an advisory agreement regarding the development of the device reported in this study.

## FUNDING INFORMATION

None.
